# Improving long-term health outcomes of preterm infants: how to implement the findings of nutritional intervention studies into daily clinical practice

**DOI:** 10.1007/s00431-021-03950-2

**Published:** 2021-01-30

**Authors:** Charlotte A. Ruys, Monique van de Lagemaat, Joost Rotteveel, Martijn J. J. Finken, Harrie N. Lafeber

**Affiliations:** 1 Department of Pediatrics/Neonatology, Emma Children’s Hospital, Amsterdam UMC, VU University Amsterdam, PO Box 7057, 1007 MB Amsterdam, The Netherlands; 2 Department of Pediatric Endocrinology, Emma Children’s Hospital, Amsterdam UMC, VU University Amsterdam, Amsterdam, The Netherlands

**Keywords:** Preterm birth, Early nutrition, Postnatal growth restriction, Protein-to-energy ratio

## Abstract

Preterm-born children are at risk for later neurodevelopmental problems and cardiometabolic diseases; early-life growth restriction and suboptimal neonatal nutrition have been recognized as risk factors. Prevention of these long-term sequelae has been the focus of intervention studies. High supplies of protein and energy during the first weeks of life (i.e., energy > 100 kcal kg^−1^ day^−1^ and a protein-to-energy ratio > 3 g/100 kcal) were found to improve both early growth and later neurodevelopmental outcome. Discontinuation of this high-energy diet is advised beyond 32–34 weeks postconceptional age to prevent excess fat mass and possible later cardiometabolic diseases. After discharge, nutrition with a higher protein-to-energy ratio (i.e., > 2.5–3.0 g/100 kcal) may improve growth and body composition in the short term.

*Conclusion*: Preterm infants in their first weeks of life require a high-protein high-energy diet, starting shortly after birth. Subsequent adjustments in nutritional composition, aimed at achieving optimal body composition and minimizing the long-term cardiometabolic risks without jeopardizing the developing brain, should be guided by the growth pattern. The long-term impact of this strategy needs to be studied.

**Table:** 

**What is Known:** *• Preterm infants are at risk for nutritional deficiencies and extrauterine growth restriction.* *• Extrauterine growth restriction and suboptimal nutrition are risk factors for neurodevelopmental problems and cardiometabolic disease in later life.*	
**What is New:** *• Postnatally, a shorter duration of high-energy nutrition may prevent excess fat mass accretion and its associated cardiometabolic risks and an early switch to a protein-enriched diet should be considered from 32-34 weeks postconceptional age.* *• In case of formula feeding, re-evaluate the need for the continuation of a protein-enriched diet, based on the infant’s growth pattern.*

**What is Known:**

*• Preterm infants are at risk for nutritional deficiencies and extrauterine growth restriction.*

*• Extrauterine growth restriction and suboptimal nutrition are risk factors for neurodevelopmental problems and cardiometabolic disease in later life.*

**What is New:**

*• Postnatally, a shorter duration of high-energy nutrition may prevent excess fat mass accretion and its associated cardiometabolic risks and an early switch to a protein-enriched diet should be considered from 32-34 weeks postconceptional age.*

*• In case of formula feeding, re-evaluate the need for the continuation of a protein-enriched diet, based on the infant’s growth pattern.*

## Introduction

Over the past decades, the incidence of preterm birth (i.e., before 37-week gestation) has increased from 7.2% in 1990 to 8.6% in 2010 in developed countries, with a relatively stable proportion of 15% very preterm births (i.e., before 32-week gestation) [1–4]. Meanwhile, owing to improvements in perinatal care, neonatal mortality after very preterm birth has decreased [5]. Nonetheless, the numbers with neonatal morbidities have remained unchanged or have even increased over time [5, 6]. Similar observations were made for long-term cognitive and motor impairments [7, 8]. Preterm birth has also been associated with later cardiometabolic risks [9], which emphasizes the importance of strategies that aim to improve short- and long-term outcomes after very preterm birth.

Early-life growth and nutrition may modulate these risks. This review provides a brief summary of findings from nutritional intervention studies in preterm infants and gives practical suggestions for their nutritional management from birth to 6 months corrected age (CA; i.e., after term age).

## Growth after preterm birth and its impact on neurodevelopment and cardiometabolic risks

Very preterm infants (i.e., gestational age < 32 weeks) or very low birth weight infants (VLBW; i.e., birth weight < 1500 g) are at risk for suboptimal postnatal growth during their extrauterine “third trimester” due to a combination of feeding problems, gut immaturity, and high nutritional demands (i.e., due to acute illnesses and rapid brain growth) [10]. Therefore, the first weeks after birth are characterized by a discrepancy between the nutritional demands and the nutritional supplies, which is particularly evident among ill preterm infants [11]. Accumulating deficits of energy and macronutrients result in extrauterine growth restriction (EUGR) in 33 to 90% of VLBW infants, depending on the definition [12–14]. There is controversy about the definition of EUGR, including cutoff level (< − 2 SDS vs < 10th percentile) and age of assessment (term age vs 36 weeks postconceptional age) [14–16]. In this review, all infants with weight below − 2 SDS or below the 10th percentile at term age or at 36 weeks CA were considered as having EUGR.

The third trimester of pregnancy is a period of rapid fetal brain growth and development in terms of cortical thickening, myelination, axonal development, vascularization, and cerebellar growth [17]. Preterm infants, specifically those who experienced EUGR, show delayed cortical maturation until term age [18] and impaired neurodevelopment in childhood [14–16] or at adolescence [19]. They are at increased risk for cognitive, behavioral, and motor problems [20, 21]. One-fourth of them has moderate to severe impairments [22]. The reduction in intelligence quotient score associated with prematurity has been estimated at 0.86 SD [8]. Preterm infants experiencing a more rapid postnatal growth generally have a more favorable neurodevelopmental outcome [23, 24].

Furthermore, preterm birth has been associated with metabolic syndrome in later life [9, 25–34], which is a combination of increased fat mass, high blood pressure, dyslipidemia, and impaired glucose tolerance [35]. The link between prematurity and metabolic syndrome may be partly explained by catch-up growth after EUGR [14, 36]. Although those born with extremely low birth weight (i.e., < 1000 g) do experience accelerated postnatal growth, they still remain smaller and lighter throughout childhood than those term born with inconsistent findings with regard to body composition [37]. Nonetheless, catch-up growth accompanied by fat mass accretion exceeding linear growth predisposes to infant adiposity, and subsequent tracking of fat mass from infancy into childhood and adulthood predisposes to metabolic syndrome [38]. In line with these findings, more rapid weight-for-length increments during the first 3 months after preterm birth have been associated with metabolic syndrome components at age 18–24 years [39].

## Improvement of nutritional status and postnatal growth

The high nutritional needs of preterm infants can only be met by a diet rich in carbohydrates, protein, and fat, starting shortly after birth. This window of opportunity has been the focus of multiple nutritional intervention studies [40, 41]. Most of these studies aim to achieve postnatal growth, fat mass excretion, and neurodevelopment similar to intrauterine norms. However, long-term follow-up of nutritional intervention studies is scarce [42]. Despite major improvements in the nutritional composition for preterm infants over the past decades [43, 44], cumulative nutritional deficits and EUGR are still common at the time of hospital discharge [45]. Therefore, nutritional interventions after discharge may also contribute to improving long-term outcomes [40, 41].

## Translating nutritional research into daily practice

**Fig. 1:**
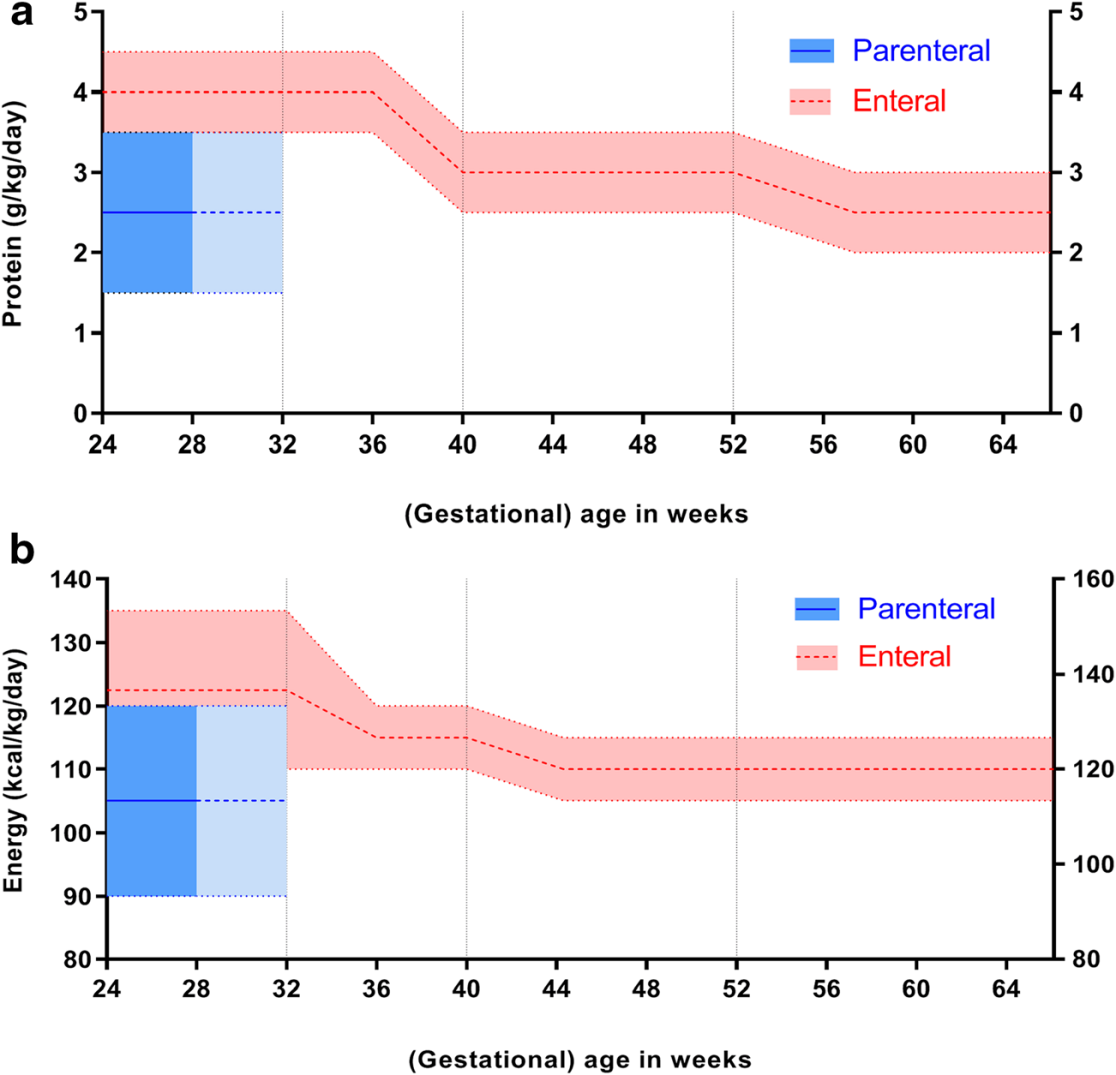
Recommendations for protein intake (**a**) and energy intake (**b**) from birth until 6 months corrected age represented as means with ranges (colored dotted lines) from parenteral (blue) and enteral (red) nutrition. Vertical dotted lines represent the “transition period” from 32 to 34 weeks postconceptional age to term age when energy intake may be (gradually) lowered to 115 kcal kg^−1^ day^−1^ with a protein intake of ≥ 3 g kg^−1^ day^−1^, provided that growth is age appropriate (i.e., 10–15 g kg^−1^s day^−1^ for at least 1 week) [45, 47]. Total protein intake from combined parenteral and enteral nutrition should not exceed 4.5 g kg^−1^ day^−1^; total energy intake from combined parenteral and enteral nutrition should not exceed 135 kcal kg^−1^ day^−1^

**Fig. 2:**
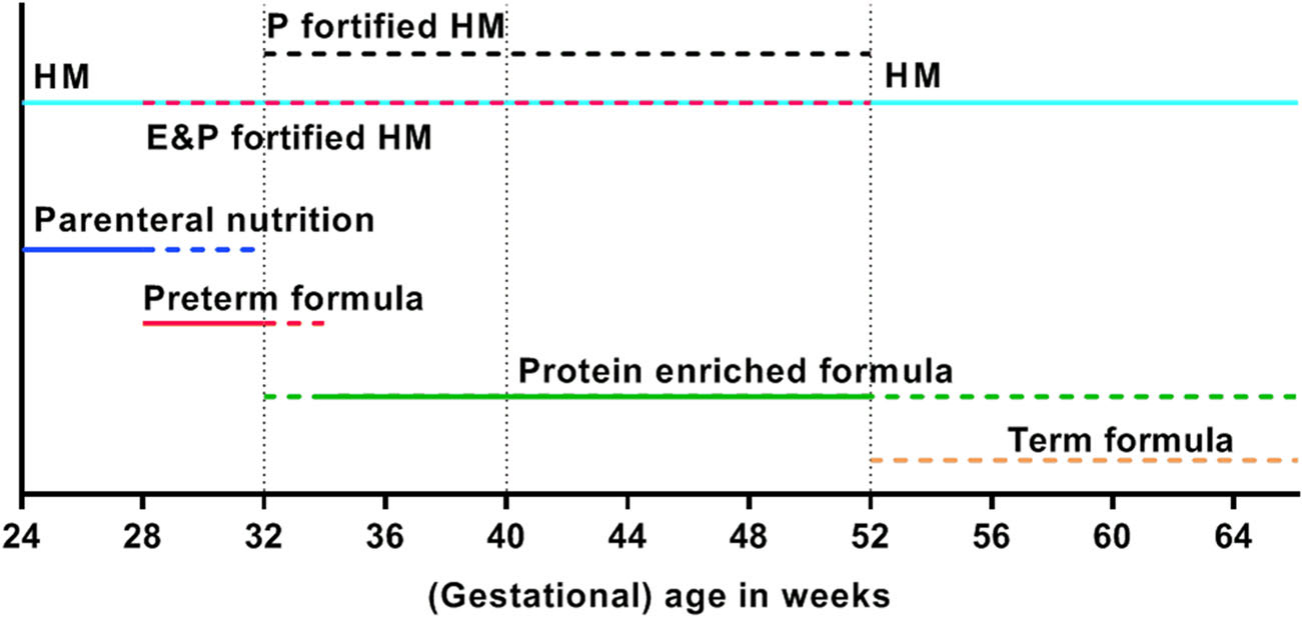
Recommendations for the type of nutrition (colored horizontal lines) from birth until 6 months corrected age. Vertical dotted lines represent “transition periods” from 32 to 34 weeks postconceptional age to term age when energy intake may be (gradually) lowered to 115 kcal kg^−1^ day^−1^ with a protein intake of ≥ 3 g kg^−1^ day^−1^, provided that growth is age appropriate (i.e., 10–15 g kg^−1^ day^−1^ for at least 1 week) [45, 47]

Based on the available evidence [46–49], we propose practical suggestions for the nutritional management of very preterm infants from (very) preterm birth until 32–34 weeks postconceptional age (part A), from 32 to 34 weeks postconceptional age until term age (part B), and from term age until 6 months CA (part C). Figures 1 and 2 provide a graphical representation of these practical suggestions and are based on the total protein and energy intakes that may theoretically be needed by these (very) preterm infants.

### Part A: Practical nutritional suggestions for (very) preterm infants from very preterm birth (i.e., gestational age < 32 weeks) until 32–34 weeks postconceptional age

There is sufficient evidence to recommend an early initiation and a rapid advancement of protein and energy intakes in very preterm infants [43, 46, 50, 51]. From 24 weeks until 28–30 weeks postconceptional age, infants are admitted to a neonatal intensive care unit and require parenteral nutrition for the first 2 to 3 weeks of life to fulfill their nutritional demands. Meanwhile, enteral nutrition should be gradually increased. Studies conducted thus far show that providing preterm infants with 1.5 g of parenteral amino acids per kilogram per day within the first 24 h after birth and increasing its stepwise to 3.5–4.0 g kg^−1^ day^−1^ is safe, and these intakes have been adhered to by current global guidelines on parenteral nutrition in VLBW infants [50, 54]. Furthermore, increased supplies of protein and energy during the first weeks of life improve neurodevelopmental outcome at 18 months of age [52, 53]. After the advancement of enteral supplies, the enteral protein intake should add up to 3.5–4.5 g kg^−1^ day^−1^ (for birth weight below 1000 g: 4.0–4.5 g kg^−1^ day^−1^; for birth weight 1000–1800 g: 3.5–4.0 g kg^−1^ day^−1^) and the energy intake to 110–135 kcal kg^−1^ day^−1^, in line with ESPGHAN recommendations (Fig. 1) [45, 47, 55]. In practice, these protein and energy intakes can be achieved with human milk (HM), either mother’s own milk or donor milk, with the addition of a breast milk fortifier and, if necessary, protein enrichment. When formula fed, these intakes can be achieved with a preterm formula with sufficiently high-protein content. A Cochrane review demonstrates that a formula with a protein content of 1.7 g/100 ml already results in a modest effect on growth rates during hospital admission in preterm infants with a birth weight ≤ 1850 g [48]. Moreover, another Cochrane review states low-certainty evidence of improved weight gain during hospital admission with high (> 3 g kg^−1^ day^−1^) compared to low (< 3 g kg^−1^ day^−1^) protein intake from formula [56].

However, controversy exists on the further benefit on weight gain before discharge with a very high enteral protein intake (> 4 g kg^−1^ day^−1^) as one study describes the increase in weight gain [57], whereas others, including a recent Cochrane review, do not find any benefits [56, 58, 59]. Not only quantitative weight gain but also lean mass may be influenced by higher protein intake during the hospital stay with a higher increase in lean mass with protein intake up to 4 g kg^−1^ day^−1^ [57]. In addition, among very preterm and VLBW infants, an insufficient protein intake during the first weeks of life is associated with decreases in lean mass and resting energy expenditure in adulthood [60], while excess energy intake (i.e., an intake exceeding the amount needed for appropriate growth) has been associated with gains in fat mass [61, 62].

There is a prominent role for the use of fortified human milk (HM), either own mother’s milk or donor HM [47, 63, 64], but randomized trials comparing growth in very preterm infants fed preterm formula or HM during a hospital stay are not available [48]. Observational studies inconsistently show improved growth up to 12 months CA with preterm formula in one study [65] and no difference in growth between preterm formula or HM in another [66]. A meta-analysis of six trials shows higher (absolute and relative) fat mass at term age in very preterm infants fed formula compared to HM [67]. When comparing preterm formula to donor HM, a systematic review shows that very preterm infants fed preterm formula demonstrate faster growth before hospital discharge [49].

As shown by Cochrane reviews and several studies, multinutrient fortification of HM results in improved growth in the short term [64, 68–70], which does not sustain during the first year of life. In particular, protein supplementation to HM increases short-term rates of weight, length, and head circumference gain in preterm infants during hospital admission [68, 71, 72] without a clear evidence of a higher risk of necrotizing enterocolitis or feeding intolerance [68]. There is insufficient evidence for the effect of fat or carbohydrate supplementation to HM on growth rate [73, 74]. In addition, there is some evidence suggesting that HM might protect against metabolic syndrome [75]. This protective effect of HM might be explained by a favorable early growth pattern as compared with formula feeding [75]. Ideally, HM fortification should be individualized since it has been demonstrated that substantial inter-individual variation in macronutrient contents of (preterm) HM could explain differences in growth velocity [76]. A preterm formula can serve as an (second best) alternative if donor HM is not available or refused by parents (Table 1), in spite of a higher risk of feeding intolerance and necrotizing enterocolitis compared to donor HM [48, 49, 77, 78].

**Table 1:** Composition of different types of (preterm) infant nutrition per 100 ml

	HM term^a^	HM preterm^a^	HM fortifier	HM protein fortifier^b^	Preterm formula	Energy & protein-enriched formula	Protein-enriched formula	Standard term formula
Energy (kcal)	68	65	14–16	3.4	79–100	74–77	66–68	64–68
Protein (g)	1.0	1.5	1.0–1.2	0.8	2.6–3.0	2.0–2.1	2.0–2.2	1.3–1.4
P:E ratio	1.5	2.3	-	-	3.0–3.6	2.7–2.8	3.0–3.3	2.0–2.1
Carbohydrates (g)	7.0	7.2	1.8–2.8	0.02	7.8–9.6	7.5–7.8	6.6–7.3	6.9–7.6
Lipids (g)	4.0	3.5	-	0.001	3.9–6.7	4.0–4.1	3.4–3.7	3.4–3.6
Calcium (mg)	30	25	66–116	5.2	96–183	53–120	66–165	44–57
Phosphate (mg)	15	14	38–64	5.2	54–101	46–66	39–90	26–43
Iron (mg)	0.1	0	0–0.32	-	1.5–1.8	1.2–1.8	0.9–1.8	0.4–1.8
Vitamin D (μg)	0	0	3.0–5.0	-	1.9–7.5	1.3–3.1	1.3–7.5	1.2–1.5

Based on available products in Europe (Nenatal/Nutrilon Nutricia, HeroBaby, Humana, Nutriprem Cow&Gate, Enfamil Mead Johnson, Similac Abbott)

^a^Based on Gidrewicz et al. [98] and Boyce et al. [99]

^b^Per sachet of 1 g. (E&)P-enriched formula, (energy&) protein-enriched formula; *HM*, human milk; *HMF*, human milk fortifier; *HMPF*, human milk protein fortifier; *P:E ratio*, protein-to-energy ratio; *STF*, standard term formula

In summary, in clinical practice, initial parenteral nutrition may be rapidly increased with regard to protein intake from 1.5 g kg^−1^ day^−1^ during the first 24 h to 3.5–4.0 g kg^−1^ day^−1^ thereafter. With the advancement of enteral nutrition, it is advised to use (donor) HM with multinutrient or protein fortification as the first choice of enteral feeding and preterm formula as a second-best alternative. Total enteral and parenteral intake should aim at an energy intake of 110–135 kcal kg^−1^ day^−1^ and a protein intake of 4.0–4.5 g kg^−1^ day^−1^ for preterm infants with a birth weight below 1000 g and a protein intake of 3.5–4.0 g kg^−1^ day^−1^ for preterm infants with a birth weight of 1000–1800 g.

### Part B: Practical nutritional suggestions from 32–34 weeks postconceptional age to term age

In addition to the ESPGHAN recommendations, we suggest that nutritional decision-making from 32–34 weeks postconceptional age is guided by the infant’s growth pattern. From that age onwards, the energy intake may be lowered from 135 to around 115 kcal kg^−1^ day^−1^ (Fig. 1), provided that growth is 10–15 g kg^−1^ day^−1^ for at least 1 week, similar to fetal growth [45, 47]. Beyond 32–34 weeks postconceptional age, a high-caloric diet may lead to excessive fat mass gain [79] and a switch to a mainly protein-enriched diet might be necessary to ensure optimal lean mass relative to fat mass accretion. While gradually tapering the amount of energy from 32–34 weeks postconceptional age onward, high-protein supplies (≥ 3 g kg^−1^ day^−1^) resulting in a higher protein-to-energy (P:E) ratio must be maintained to meet the requirements for linear growth and brain development [47, 68]. Prolongation of human milk fortification with protein until term age or use of a protein- but not energy-fortified formula may be considered during this period. To date, there are no meta-analyses or systematic reviews available to support this suggestion.

### Part C: Practical nutritional suggestions from term age until 6 months CA

From term age onwards, we recommend maintaining the intake of energy and protein at ~ 110 kcal kg^−1^ day^−1^ and ~ 3 g kg^−1^ day^−1^, respectively (Fig. 1) [47]. Preterm infants who are completely HM fed will most likely drink directly from the breast by this time, making fortification impractical. The protein and energy contents of HM are lower than recommended. Therefore, we suggest close monitoring of the infant’s growth pattern. Protein fortification of HM beyond term age should only be considered if steady growth has not yet been established.

In preterm infants who are formula fed, the recommended protein and energy intakes are covered by (energy-and-)protein-enriched (so-called postdischarge) formulas (Table 1 and Fig. 1). The ESPGHAN advises to use such formulas up until 40 to 52 weeks postconceptional age (i.e., until term age to 3 months CA) and, thereafter, to switch to standard term formula [47]. To date, there is no compelling evidence for an effect of nutrient-enriched formulas on growth or neurodevelopment [46, 80]. Nevertheless, a higher P:E ratio tends to be associated with improved growth and body composition during the first 6 months [46, 81–86], albeit not unequivocally [87–90]. Long-term follow-up data among infants born very preterm and/or with VLBW show that the short-term benefits on body composition of a protein-enriched formula (with a P:E ratio of 2.52 g per 100 kcal) after discharge were no longer present at age 8 years [91]. In addition, their neurodevelopmental outcomes at 18 and 24 months CA and at age 8 years were no different from those fed a standard formula after discharge [80, 89, 92, 93]. However, post hoc analyses show that preterm infants with limited postnatal weight gain during the first 6 months after term age receiving protein-enriched formula were taller and had more lean mass at age 8 years [91]. Because of the large proportion of preterm infants that are growth-restricted at term age [12, 13], we suggest a continuation of an (energy-and-)protein-enriched formula until at least 3 months CA in formula-fed preterm infants. However, this nutritional suggestion should be balanced against observations in term-born children, which suggest that excess protein intake during the first 2 years of life may predispose to later obesity [94]. Therefore, caution is warranted with the use of (energy-and-)protein-enriched formulas beyond 3 months CA. Evaluation of the growth pattern should guide the decision whether or not to continue protein-enriched formulas beyond 3 months CA up to a maximum of 6 months CA. This is only possible by using an individual approach with frequent follow-up of weight, length, head circumference, and, preferably, an estimate of body composition. Growth is preferably evaluated with postnatal growth curves for preterm infants [95, 96]. Factors such as the degree of IUGR at preterm birth and early postnatal growth (deviation from the norm) will guide the decision to fortify nutrition with extra energy and/or protein or to change to standard term formula or human milk without fortification sooner.

## Conclusions

Adequate supplies of nutrients shortly after preterm birth reduce the risk of suboptimal postnatal growth and, consequently, the risk of adverse neurodevelopmental outcome. A nutritional switch from a high-energy and high-protein diet to a high-protein-only diet should be considered during hospital admission from a postconceptional age of 32–34 weeks onwards. Human milk is the feeding of choice for preterm infants. In case of formula feeding, the continuation of protein-enriched formula should be reconsidered at 3 months CA based on the infant’s growth pattern [97]. More individualized nutritional care seems warranted. These nutritional suggestions may contribute to acknowledging the in-hospital and postdischarge periods as a continuum instead of separate entities, resulting in healthier early growth and a reduction of the associated risks of neurodevelopmental problems and cardiometabolic diseases.

## Data Availability

Not applicable.

## Abbreviations

AGA: Appropriate-for-gestational-age
CA: Corrected age
ESPGHAN: European Society for Paediatric Gastroenterology, Hepatology, and Nutrition
EUGR: Extrauterine growth restriction
HM: Human milk
P:E ratio: Protein-to-energy ratio
SGA: Small-for-gestational-age
VLBW: Very low birth weight
